# Exosomes and Cardiovascular Protection

**DOI:** 10.1007/s10557-016-6698-6

**Published:** 2016-10-29

**Authors:** Sean M. Davidson, Kaloyan Takov, Derek M. Yellon

**Affiliations:** The Hatter Cardiovascular Institute, 67 Chenies Mews, WC1E 6HX, London, UK

**Keywords:** Exosomes, Microvesicles, Microparticles, Stem cells, Cardioprotection

## Abstract

Most, if not all, cells of the cardiovascular system secrete small, lipid bilayer vesicles called exosomes. Despite technical challenges in their purification and analysis, exosomes from various sources have been shown to be powerfully cardioprotective. Indeed, it is possible that much of the so-called “paracrine” benefit in cardiovascular function obtained by stem cell therapy can be replicated by the injection of exosomes produced by stem cells. However, exosomes purified from plasma appear to be just as capable of activating cardioprotective pathways. We discuss the potential roles of endogenous exosomes in the cardiovascular system, how this is perturbed in cardiovascular disease, and evaluate their potential as therapeutic agents to protect the heart.

## Introduction

### What Are Exosomes?

**Fig. 1:**
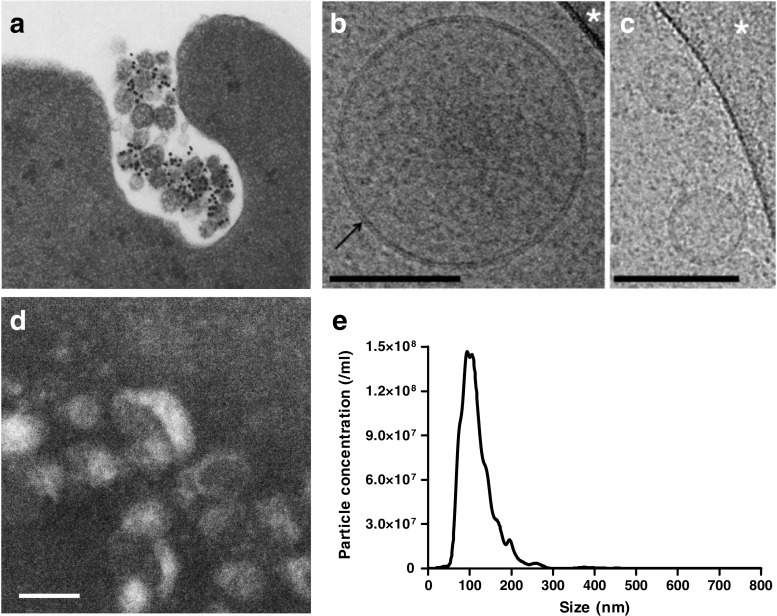
**a** Fusion of multivesicular endosomes with the plasma membrane of sheep reticulocytes and their release by exocytosis as exosomes. The gold-labelled antibodies against transferrin receptors bind to 50-nm vesicles which are inside openings of 300–800 nm in diameter. Reprinted with permission from Pan et al. [3]. **b**, **c** Cryo-electron microscopy of EVs from pure plasma. Spherical EVs embedded in a thin film of frozen platelet-free plasma. EV diameters are 185 nm in (**b**) and 45 nm and 60 nm in (**c**). The lipid bilayer at the periphery of EVs is resolved in two dark lines 4 nm apart (arrow in **b**). The granular aspect of the background is due to the high protein content of plasma. Scale bars: 100 nm. Reprinted with permission from [14]. **d** Transmission electron microscopy of exosome enriched from rat plasma by differential ultracentrifugation. Scale bar: 100 nm. **e** Size distribution exosome produced by primary adult rat cardiomyocytes as determined by nanoparticle tracking analysis

The term “exosome” was first used to describe sub-micron sized lipid vesicles released from cells, in 1981 [1]. Subsequently, the term was used more specifically to refer to ~50 nm diameter vesicles containing transferrin receptors released from maturing blood reticulocytes (Fig. 1a) [2, 3]. For many years afterwards, exosomes were considered to be purely a means for the cell to shed excess proteins. However, the demonstration in 2007 that they contained miRNA that could be transferred between cells [4], stimulated great interest in their potential as both biomarkers, and as therapeutic agents.

Most, if not all, types of cell release exosomes of 50–150 nm diameter. These are produced initially by intraluminal vesiculation within endosomal multivesicular bodies, and are referred to as exosomes when they are released from the cell. As such, they differ from microvesicles which are released by shedding of plasma membrane and are typically 100 nm - 1 μm in diameter. However, most currently used techniques are unable to obtain completely pure exosomes, and provide a mixture of exosomes, microvesicles and other extracellular vesicles such as apoptotic bodies [5, 6]. It has therefore been suggested that experimentally used isolates of cellular vesicles should be referred to conservatively as “extracellular vesicles” (EVs) or small EVs (sEVs) [5].

Commonly used techniques to isolate sEVs from blood or tissue culture medium include ultracentrifugation, precipitation, affinity-isolation and size-exclusion chromatography [7]. However, as mentioned, none of these methods are perfect and lead to co-isolation of sEVs containing some degree of microvesicles [5, 6]. Density gradient purification may be used to increase the purification of lighter vesicles from more dense proteins, but is time-consuming and low throughput. Furthermore, since other blood components such as lipoproteins, protein macromolecular complexes and plasma proteins can be of a similar size and even similar density to exosomes they will still contaminate the isolated vesicles to some degree. Similarly, when isolating sEVs from cultured cells, it is important to use serum-free medium or culture cells with serum that has been pre-cleared of sEVs, in order to reduce contamination by EVs and other serum components. Exosome-free serum is available commercially or can be prepared by overnight ultracentrifugation [8]. When using serum-free medium, an important additional consideration is the health of the cells, since cells undergoing apoptosis will fragment, releasing apoptotic bodies that can contaminate the purified vesicles.

Given the steps that must be taken in order to achieve sEV populations of reasonable purity, it is challenging to obtain yields of vesicles sufficient for downstream biochemical experiments. It can be calculated that, theoretically, a single exosome of 100 nm diameter contains ~1500 proteins (Table 1). Therefore, in a completely pure preparation of all ~10^10^ plasma exosomes in 1 ml of blood [9, 10], the maximum yield is expected to be just ~1 μg protein. Exosome concentration in conditioned, tissue-culture medium is typically much lower, meaning that litres of medium may be required to obtain sufficient protein for analysis. In terms of miRNA, Chevillet et al. determined that on average, there was far less than one molecule of a given miRNA per exosome, and that on average, over 100 exosomes would need to be examined to observe one copy of a given abundant miRNA [11]. On the other hand, PCR-based techniques greatly increase the sensitivity of miRNA or mRNA analysis.

**Table 1:** Calculation of the number of proteins per a typical exosome and cell

	Spherical exosome	Cube-shaped cell
Diameter / length	100 nm	15 μm
Volume	0.0005 μm^3^	3375 μm^3^
Density	1.15 g/ml	1.03 g/ml
Mass (volume x density)	6.0 × 10^−7^ ng	3.48 ng
Mass of protein (20 % of total^a^)	1.2 × 10^−7^ ng	0.70 ng
Number of proteins^a,b^	1390	8.0 × 10^9^
Number of exosomes/cells required for 1 μg of protein	8.3 × 10^9^	1430

^a^Assumptions made are that protein accounts for 20 % of the total exosome mass and that the average protein mass is 52 kDa, as has been determined to be the case for eukaryotic cells [60]

^b^Number of proteins = mass of proteins / 52,000 x Avogadro’s constant

Proteins from the Endosomal Sorting Complexes Required for Transport (ESCRT) machinery are involved in exosome release and commonly found associated with exosomes. However, since inhibition of key ESCRT proteins does not completely eliminate exosome production, ESCRT-independent mechanisms are also believed to be involved [12] (reviewed in [13]). Since ceramide is enriched in exosomes and was proposed to be involved in their intraluminal formation, neutral sphingomyelinase inhibitors have been used to inhibit exosome production [12]. However care must be taken in interpreting experiments, since these inhibitors are unlikely to be specific for exosome release [13].

The use of flow cytometry is challenging, even for the detection of microvesicles [14]. Since they are smaller even than the wave-length of light, specialized techniques have been developed to quantify and visualize exosomes. In particular due to their small size, flow cytometry is not applicable, and techniques such as nanoparticle tracking analysis (Fig. 1e), dynamic light scattering, or atomic force microscopy must be used to ascertain their size-range and concentration [7]. Transmission electron micrography or cryo-electron microscopy is useful to demonstrate the vesicular nature of the particles (Fig. 1b-d) [15]. Exosomes-associated proteins such as those involved in the ESCRT machinery, tetraspanins (e.g.: CD9, CD63, CD81), and HSP70 are commonly used as protein markers to further verify sEV identity as exosomes, although recent evidence suggests that some of these markers are not as specific for exosomes as once thought [5]. MVs can be further characterized by their expression of surface membrane markers which reflect their cell type of origin. It is important to note that, due to their endosomal origin, many exosomes do express similar membrane markers, and it can be more difficult to identify their cell type of origin [14]. However, as the contents of EVs typically reflect the contents of the cell of origin, profiling of the EV constituents can potentially be informative in the identification of biomarkers.

### What Are the Roles of Microvesicles and Exosomes from Cells of the Cardiovascular System?

Both microvesicles and exosomes may have roles in diabetes, cardiovascular disease, endothelial dysfunction, coagulopathies, and polycystic ovary syndrome [7]. For example, elevated levels of circulating microvesicles of endothelial origin and with procoagulant potential are found in the blood of patients with acute coronary syndromes [16]. Measuring endothelial microvesicles as an indicator of endothelial dysfunction can help to identify patients vulnerable to coronary heart disease [17].

The blood of healthy individuals contains enormous numbers of sEVs, estimated to be on the order of 10^10^ vesicles per ml of plasma [9, 10]. These are believed to originate mainly from platelets and erythrocytes, but lymphocytes, endothelial cells and parenchymal cells also contribute. Arraud et al. used a method of cryo-electron microscopy which preserves the structure of EVs in solution and demonstrated that EVs are primarily spherical with a diameter ~ 200 nm, although they also observed larger, tubular structures of unknown origin (Fig. 1b,c) [14]. By sedimenting and quantifying vesicles onto electron microscope grids, they arrived at a conservative estimate of 5 × 10^7^ / ml sEVs in platelet-free plasma. By examining the expression of surface marker proteins they concluded that similar numbers of vesicles were derived from platelets and erythrocytes [14]. Given the high numbers of sEVs in the plasma of healthy individuals, it is important to determine whether they have functions other than simply waste disposal. Evidence from the literature supporting the role of EVs of different origin in cardiovascular disease is summarized in Table 2 and will be discussed further below.

**Table 2:** Roles of EVs released from the cells of the cardiovascular system

Cell/organ of the vesicular origin	Source	Type of EVs	Cargo mediator	Function	References
A: *Roles of EVs originating from different cells of the cardiovascular system*					
Endothelial cells	HUVECs, mouse H5V cell line	Microvesicles, exosomes	miRNA-143, miRNA-145	Endothelial cells subjected to shear stress or overexpressing Krüppel-like Factor 2 (activated by shear stress) have increased expression of miRNA-143 and miRNA-145 which are secreted in the extracellular vesicle fraction and transferred to smooth muscle cells. This was atheroprotective in a mouse model. Procedure for microvesicle isolation followed, although authors noted that majority of vesicles are reminiscent of exosomes.	[37]
Endothelial cells	Human microvascular endothelial cell line	Exosomes	miRNA-214	Increased migration and tube formation of recipient endothelial cells abolished by depletion of miRNA-214 by antagomir. In mice in vivo angiogenesis was increased in implanted matrigel plugs loaded with endothelial cell-derived exosomes but not in plugs loaded with exosomes isolated from antagomir-treated endothelial cells.	[38]
Cardiomyocytes	HL-1 cell line	Exosomes	Not determined	Growth factor (TGF-β2, PDGF-BB) treatment affected messenger RNA contents of exosomes secreted by cardiomyocytes. No function reported.	[26]
Cardiomyocytes	Rat (neonatal)	Exosomes	GLUT1, GLUT4	Glucose deprivation of neonatal rat cardiomyocytes increased production of exosomes carrying GLUT1 and GLUT4 transporters which were transferred to rat cardiac microvascular endothelial cells to stimulate uptake of glucose and glycolysis.	[27]
Cardiomyocytes	H9C2 cell line	Exosomes	Not determined	Glucose deprivation increases production of exosomes in cardiomyocytes which can be transferred to HUVECs and induce transcriptional changes, proliferation and tube formation in vitro. Differential protein and miRNA content demonstrated under normal conditions or glucose deprivation which may account for these effects.	[28]
Platelets	Human	Microparticles	Lipid growth factors	Increased survival, proliferation and migration of HUVECs reduced by treatment with activated charcoal suggesting involvement of lipid growth factor mediators.	[19]
Platelets	Human	Microparticles	VEGF, bFGF, PDGF, heparanase	Stimulation of sprouting angiogenesis in a rat aortic ring model. Increased migration of endothelial cells through a matrigel membrane. Induction of angiogenesis in vivo in implanted agarose beads containing platelet microparticles. Induction of revascularisation after chronic myocardial infarction in rats.	[20]
Platelets	Human	Microparticles	CXCR4	Increased expression of mature endothelial cell markers, adhesion and migration of angiogenic early outgrowth cells by increase in CXCR4. Reduction of neointima formation after wire injury in mice in vivo.	[22]
Blood plasma/unknown cell type	Rat, human	Exosomes	HSP70	Protection against myocardial ischaemia-reperfusion injury in vitro (isolated cardiomyocytes), ex vivo (Langendorff heart preparation) and in vivo in rats by HSP70/TLR-4-mediated activation of ERK/p38 pathway in cardiomyocytes.	[10]
Reticulocytes	Rat [2], sheep [3]	Exosomes	Transferring receptors	Disposal of transferrin receptors needed for maturation of reticulocytes.	[2, 3]
B: *EVs and cardiovascular disease*					
Blood plasma, lungs	Mice (pulmonary arterial hypertension)	Exosomes	Differential miRNA profile in healthy and diseased animals	Exosomes isolated from blood plasma or lungs of mice with pulmonary arterial hypertension induce right ventricular hypertrophy and remodelling of the pulmonary vasculature in recipient healthy mice.	[41]
Blood plasma, endothelial progenitor cells	Human	Microvesicles	miRNA-126/VEGFR2	Reduction of endothelial progenitor cell migration, increased apoptosis and increased production of ROS upon treatment with blood plasma-derived or endothelial progenitor cell-derived microvesicles from patients with uncontrolled diabetes. Microvesicles from healthy controls had opposite effects. Effects attributed to decreased levels of miRNA-126 and VEGFR2 in endothelial progenitor cells in uncontrolled diabetes.	[30]
Blood plasma (endothelial origin)	Human	Microparticles	N/A	Endothelial microparticles with procoagulant potential were elevated in patients with unstable angina and myocardial infarction. No function reported.	[16]
Platelets, erythrocytes, endothelial cells	Human	Microparticles	N/A	Rise in platelet, erythrocyte and endothelial cell microparticles with procoagulant potential after sympathomimetic stress echocardiogram in healthy but not vascular disease patients. No function identified.	[23]
Pericytes, endothelial cells	Human	Exosomes	Not determined	Pericytes stimulated with CoCl_2_ induce pro-angiogenic response in endothelial cell as shown in would healing and spinal cord tissue angiogenesis assays. These pro-angiogenic effects were ameliorated upon pharmacological inhibition of exosome secretion.	[36]
Vascular smooth muscle cells	Human	Exosomes / matrix vesicles	Calcium/phosphate deposits	Vascular smooth muscle cell-derived matrix vesicles (identified as exosomes) shown to carry mineral deposits. Calcifying conditions increased exosome production of vascular smooth muscle cells. Chronic kidney disease and atherosclerotic patients shown to have high calcifying exosome content in arteries.	[31]
Cardiomyocytes	Healthy and Goto-Kakizaki diabetic rats	Exosomes	miRNA-320	Inhibition of proliferation and migration of mouse cardiac endothelial cells upon incubation with exosomes secreted by Goto-Kakizaki (GK) rat cardiomyocytes. Exosomes isolated from healthy Wistar rats had opposite effects. miRNA-320 was upregulated in GK rats exosomes and delivered to endothelial cells inhibiting proangiogenic target genes.	[29]

The role of platelet sEVs is unclear as they appear to have both detrimental and beneficial effects [18]. They are often pro-thrombotic, but platelet-derived sEVs can also stimulate angiogenesis both in vitro and in vivo [19, 20]. Injection of platelet microvesicles into the ischaemic myocardium increased the number of functioning capillaries in a rat model of chronic myocardial ischaemia [20]. After vascular injury, platelets become activated locally, and degranulate, releasing sEVs including both exosomes and microvesicles [21]. Platelet-derived sEVs have been shown to interact with angiogenic early outgrowth cells, altering SDF-1α/CXCR4 signalling, and stimulating their maturation and re-endothelialisation [22]. Irrespective of their function, measurement of plasma microvesicle concentration may have a useful diagnostic role. For example, in the absence of coronary artery disease, the numbers of microvesicles derived from platelets and other cell types increased immediately following a stress echocardiogram, while in the presence of coronary artery disease numbers remained unchanged [23].

Cardiomyocytes have been shown to release exosomes, at least in vitro (Fig. 1e) [24, 25]. The protein and mRNA content of their exosomes varies under different culture conditions and stimulus such as oxidative stress [24, 26]. Under conditions of oxidative stress or glucose deprivation, neonatal cardiomyocytes and cardiac-like H9c2 cells release increased numbers of exosomes [27]. In co-culture with endothelial cells, these exosomes induced endothelial proliferation and angiogenesis as well as increasing glucose uptake and glycolytic activity in recipient cells [27, 28]. Interestingly, exosomes obtained from diabetic rat myocytes have not only lost their pro-angiogenic capacity, but actively inhibit angiogenesis [29]. This appears to be via exosomal transfer of miR-320 and down-regulation of its target genes (IGF-1, Hsp20 and Ets2) in recipient cardiac endothelial cells [29]. Circulating microvesicles of diabetic mice have also been found to have negative effects on the function of endothelial progenitor cells due to altered miR-126 levels [30]. Thus, a loss of exosomal function may contribute to some aspects of cardiovascular diseases.

Vascular calcification is associated with major adverse cardiovascular events. In the earliest phase of mineralization, vascular smooth muscle cells secrete exosomes which nucleate calcium phosphate crystals and promote vascular calcification [31]. This response is exacerbated in response to environmental calcium stress, suggesting that modulation of the exosome release pathway in vascular smooth muscle cells may be a novel therapeutic target for prevention of calcification.

There is increasing interest in the mechanisms of communication between cell types of the heart, and whether these can be harnessed for therapeutic purposes. For example, some drugs may work by stimulating communication of cardioprotective pathways between endothelium and cardiomyocytes [32]. Exosomes and microvesicles are an important means of intercellular communication, particularly during development and via specialized channels of communication such as the immune synapse [33, 34], but evidence is accumulating for their communication role in the cardiovascular system [7, 35]. For example, co-culture of primary endothelial cells with activated pericytes, which normally surround and communicate with them in vivo, stimulates their angiogenic properties in an exosome-dependent manner [36]. Endothelial cells control target gene expression in co-cultured smooth muscle cells by transferring sEVs enriched in miR-143/145 in response to shear stress [37]. The therapeutic potential of this novel pathway was demonstrated by injecting sEVs into atherosclerosis-prone ApoE(−/−) mice and observing a reduction in atherosclerotic lesion formation in the aorta [37]. A further benefit of endothelial cell-derived exosomes is that they stimulate migration and angiogenesis in recipient cells [7]. This effect was found to be dependent on expression of miR-214 [38]. Data such as this has increased interest in the potential for the use of sEVs and exosomes as therapeutic agents.

### What Is the Therapeutic Potential of Exosomes?

Exosomes have been found to offer therapeutic benefit in various experimental cardiovascular models (Table 3). For example, sEVs derived from adipose mesenchymal stem cells (MSC) inhibit vascular smooth muscle proliferation and migration in vitro, and when injected into mice over a 20 day period they reduced the extent of intimal hyperplasia in transplanted vein grafts [39]. Levels of inflammatory cytokines interleukin (IL)-6 and monocyte chemoattractant protein-1 (MCP-1) were also reduced. Intravenous delivery of MSC-derived exosomes also inhibited vascular remodelling and hypertension in a mouse model of hypoxia-induced pulmonary hypertension, potentially via inhibition of STAT3 signalling in pulmonary artery endothelial cells [40]. Another interesting study published very recently confirmed the potential of MSC-derived exosomes in reversing pulmonary hypertension in mice, and furthermore showed that exosomes derived from mice with monocrotaline-induced pulmonary hypertension can induce pulmonary hypertension when injected in a non-diseased animal [41]. The differential effects were attributed to the different miRNA profiles of the exosomes.

**Table 3:** Cardioprotection by cardiac-, plasma- and stem-cell-derived exosomes

Source of exosomes	Protection observed	Mechanism	Reference
Cardiac endothelial cells	Improved ejection fraction in mice after MI	Delivery of miR-126 and miR-210 to CPCs, activating prosurvival kinases and inducing a glycolytic switch	[48]
Human or rat plasma	Cardioprotection	Exosomal HSP70 activating MAPK/ERK1/2 signalling via TLR4	[10]
CD34^+ve^ haematopoietic stem cells	Angiogenic activity both in vitro and in vivo	Not determined	[44]
Embryonic stem cells (ESCs)	Enhanced neovascularization, cardiomyocyte survival, and reduced fibrosis post MI	delivery of miR-294 to c-kit^+ve^ cardiac progenitor cells	[46]
Cardiac progenitor cells (CPCs)	Stimulate the migration of endothelial cells in vitro	Matrix metalloproteinases	[49]
Cardiac progenitor cells (CPCs)	Reduced apoptosis, enhanced angiogenesis, improved ejection fraction in rat MI	Potentially via delivery of miR-210, miR-132, and miR-146a-3p	[47]
Cardiospheres (CSp-EMVs)	Primed fibroblasts, which stimulated angiogenesis and cardioprotection when injected in MI	Increased secretion of the SDF-1α and VEGF	[52]
Cardiosphere-derived cells (CDCs)	Improved cardiac function in murine MI inhibiting apoptosis and promoting proliferation of cardiomyocytes, while enhancing angiogenesis	Potentially via delivery of miR-146a	[50]
Cardiosphere-derived cells (CDCs)	Decrease apoptosis and fibrosis and improved function in a doxorubicin-induced cardiomyopathy	Not determined	[51]
Mesenchymal stem cells (MSCs)	Reduced infarct size and improved recovery in a mouse MI	Akt and GSK-3β signalling pathways	[54–56]

Injection of stem cells into the myocardium is able to protect the heart against ischaemia and reperfusion injury as well as improving cardiac function after injury, via repair and possibly regeneration. Transplantation of human CD34^+ve^ hematopoietic stem cells to ischemic tissues induces neovascularization in preclinical models and has been associated with reduced angina and improved exercise time in phase 2 clinical trials. However, the benefits obtained in such experiments frequently appear to depend more on paracrine signalling effects rather than on myocardial cell engraftment of the stem cells [42]. sEVs, and exosomes in particular, have been hypothesized to mediate some of this paracrine benefit [43]. Indeed, CD34^+ve^-secreted exosomes have angiogenic activity both in vitro and in vivo [44]. Interestingly, despite the benefit of CD34^+ve^ exosomes observed on angiogenesis, CD34^+ve^ hematopoietic stem cells were not cardioprotective when administered after acute myocardial infarction unless engineered to express sonic hedgehog (Shh) [45].

The injection of exosomes derived from embryonic stem cells into infarcted mouse hearts enhanced neovascularization, cardiomyocyte survival, and reduced fibrosis, which was linked to the delivery of miR-294 to c-kit^+ve^ cardiac progenitor cells in the myocardium and consequent increase in their regenerative activity [46]. A further benefit of exosomes in this example, is the lack of carcinogenic potential that they represent in comparison to embryonic stem cells.

Exosomes from cardiac progenitor cells (CPCs) can be obtained from atrial appendage explants from patients undergoing heart valve surgery [47]. When these exosomes were administered to rats with myocardial infarction, there was less cardiomyocyte apoptosis, enhanced angiogenesis, and improved LV ejection fraction [47]. No matter which type of stem cells is used, the majority of cells die or are lost shortly after implantation. In order to improve their resistance to hypoxia, CPCs were co-delivered with a non-viral, minicircle plasmid carrying HIF1 (MC-HIF1) [48]. In vitro experiments suggested that this caused endothelial cells to overexpress HIF1, and produce exosomes with higher contents of miR-126 and miR-210 that were actively internalized by recipient CPCs, activating prosurvival kinases and inducing a glycolytic switch [48]. Conversely, exosomes derived from CPCs have also been shown to stimulate the migration of endothelial cells [49].

Exosomes secreted by cardiosphere-derived cells (CDCs), improved cardiac function when delivered in murine models of myocardial infarction, inhibiting apoptosis and promoting proliferation of cardiomyocytes, while enhancing angiogenesis [50]. Part of the benefit could be replicated by the administration of miR-146a, which was enriched in the exosomes [50]. Furthermore, systemic delivery of these exosomes was able to decrease apoptosis and fibrosis in a mouse model of doxorubicin-induced dilated cardiomyopathy [51].

In a different approach, exosomes from cardiospheres (CSp-EMVs), have been used to prime fibroblasts in vitro. This exposure was found to increase fibroblast secretion of the pro-angiogenic factors, stromal cell-derived factor 1 and vascular endothelial growth factor [52, 53]. When injected into the hearts of rats in a chronic model of myocardial infarction, the primed fibroblasts were found to stimulate significant angiogenesis and cardioprotection [52].

Based on the above evidence that stem cell exosomes provide long-term benefit by stimulating cardioprotective pathways, experiments were performed to determine whether they could confer cardioprotection acutely. Indeed, exosomes from mesenchymal stem cells (MSCs) reduced infarct size and improved recovery in a mouse model of myocardial ischemia/reperfusion injury, via a mechanism that appeared to involve Akt and GSK-3β [54–56]. Given the high number of exosomes in blood, it was hypothesized that these may also exert cardioprotective properties [7, 35, 57]. A population of sEVs enriched in exosomes was purified from blood and shown to protect rat hearts and cardiomyocytes against acute ischaemia and reperfusion injury when administered either in vivo or in vitro [10]. Protection was activated by HSP70 present in the exosomal membrane interacting with TLR4 receptor in cardiomyocytes, which lead to activation of the MAPK/ERK1/2 signalling pathway [10]. It is not yet known whether this process is perturbed under disease situations.

Some cardioprotective strategies have been shown to increase the number of sEVs in the blood. For example, ischaemic preconditioning of an isolated, perfused rat heart increases the release of HSP-60 containing vesicles [58]. Furthermore, remote ischaemic preconditioning doubles the numbers of exosomes in the blood [10] although this has not been associated with cardioprotective mechanism of remote ischaemic conditioning [10].

### Future Perspectives

Many questions remain in this new and exciting field of exosome research. Not least are technological aspects of how to obtain more pure populations of exosomes. Biologically, the normal role of circulating exosomes is unclear. The ability to study the in vivo role of exosomes will be greatly facilitated by the discovery of a specific inhibitor of the production or uptake. In terms of therapy, a better understanding of exosome pharmacokinetics and pharmacodynamics is essential. Ideally, a means of targeting them to the heart or target cells will be required, in order to avoid the requirement for intramyocardial delivery or the non-specific approach of systemic delivery. Despite these hurdles, there remains great excitement about the potential for exosomes as therapeutic agents in cardiovascular diseases, and there are already blueprints in place for the administration of extracellular vesicles based therapeutics in clinical trials [59]. However, it is important that the excitement for translation does not cloud the fact that there still remains a great deal to learn about these tiny circulating vesicles.

## References

[1] Trams EG, Lauter CJ, Salem N, Heine U (1981). Exfoliation of membrane ecto-enzymes in the form of micro-vesicles. Biochim Biophys Acta.

[2] Harding C, Heuser J, Stahl P (1984). Endocytosis and intracellular processing of transferrin and colloidal gold-transferrin in rat reticulocytes: demonstration of a pathway for receptor shedding. Eur J Cell Biol.

[3] Pan BT, Teng K, Wu C, Adam M, Johnstone RM (1985). Electron microscopic evidence for externalization of the transferrin receptor in vesicular form in sheep reticulocytes. J Cell Biol.

[4] Valadi H, Ekstrom K, Bossios A, Sjostrand M, Lee JJ, Lotvall JO (2007). Exosome-mediated transfer of mRNAs and microRNAs is a novel mechanism of genetic exchange between cells. Nat Cell Biol.

[5] Kowal J, Arras G, Colombo M, Jouve M, Morath JP, Primdal-Bengtson B (2016). Proteomic comparison defines novel markers to characterize heterogeneous populations of extracellular vesicle subtypes. Proc Natl Acad Sci U S A.

[6] Sodar BW, Kittel A, Paloczi K, Vukman KV, Osteikoetxea X, Szabo-Taylor K (2016). Low-density lipoprotein mimics blood plasma-derived exosomes and microvesicles during isolation and detection. Sci Rep.

[7] Lawson C, Vicencio JM, Yellon DM, Davidson SM (2016). Microvesicles and exosomes: new players in metabolic and cardiovascular disease. J Endocrinol.

[8] Shelke GV, Lasser C, Gho YS, Lotvall J. Importance of exosome depletion protocols to eliminate functional and RNA-containing extracellular vesicles from fetal bovine serum. J Extracell Vesicles. 2014; 3. 10.3402/jev.v3.24783 PMC4185091 25317276

[9] Dragovic RA, Gardiner C, Brooks AS, Tannetta DS, Ferguson DJ, Hole P (2011). Sizing and phenotyping of cellular vesicles using nanoparticle tracking analysis. Nanomedicine : Nanotechnology, Biology, and Medicine.

[10] Vicencio JM, Yellon DM, Sivaraman V, Das D, Boi-Doku C, Arjun S (2015). Plasma exosomes protect the myocardium from ischemia-reperfusion injury. J Am Coll Cardiol.

[11] Chevillet JR, Kang Q, Ruf IK, Briggs HA, Vojtech LN, Hughes SM (2014). Quantitative and stoichiometric analysis of the microRNA content of exosomes. Proc Natl Acad Sci U S A.

[12] Trajkovic K, Hsu C, Chiantia S, Rajendran L, Wenzel D, Wieland F (2008). Ceramide triggers budding of exosome vesicles into multivesicular endosomes. Science.

[13] Kowal J, Tkach M, Thery C (2014). Biogenesis and secretion of exosomes. Curr Opin Cell Biol.

[14] Arraud N, Linares R, Tan S, Gounou C, Pasquet JM, Mornet S (2014). Extracellular vesicles from blood plasma: determination of their morphology, size, phenotype and concentration. J Thromb Haemost.

[15] Thery C, Amigorena S, Raposo G, Clayton A. Isolation and characterization of exosomes from cell culture supernatants and biological fluids. Curr Protoc Cell Biol. 2006; Chapter 3:Unit 3.22. 10.1002/0471143030.cb0322s30 18228490

[16] Mallat Z, Benamer H, Hugel B, Benessiano J, Steg PG, Freyssinet JM (2000). Elevated levels of shed membrane microparticles with procoagulant potential in the peripheral circulating blood of patients with acute coronary syndromes. Circulation.

[17] Nozaki T, Sugiyama S, Koga H, Sugamura K, Ohba K, Matsuzawa Y (2009). Significance of a multiple biomarkers strategy including endothelial dysfunction to improve risk stratification for cardiovascular events in patients at high risk for coronary heart disease. J Am Coll Cardiol.

[18] Martinez MC, Andriantsitohaina R (2011). Microparticles in angiogenesis: therapeutic potential. Circ Res.

[19] Kim HK, Song KS, Chung JH, Lee KR, Lee SN (2004). Platelet microparticles induce angiogenesis in vitro. Br J Haematol.

[20] Brill A, Dashevsky O, Rivo J, Gozal Y, Varon D (2005). Platelet-derived microparticles induce angiogenesis and stimulate post-ischemic revascularization. Cardiovasc Res.

[21] Heijnen HF, Schiel AE, Fijnheer R, Geuze HJ, Sixma JJ (1999). Activated platelets release two types of membrane vesicles: microvesicles by surface shedding and exosomes derived from exocytosis of multivesicular bodies and alpha-granules. Blood.

[22] Mause SF, Ritzel E, Liehn EA, Hristov M, Bidzhekov K, Muller-Newen G (2010). Platelet microparticles enhance the vasoregenerative potential of angiogenic early outgrowth cells after vascular injury. Circulation.

[23] Augustine D, Ayers LV, Lima E, Newton L, Lewandowski AJ, Davis EF (2014). Dynamic release and clearance of circulating microparticles during cardiac stress. Circ Res.

[24] Malik ZA, Kott KS, Poe AJ, Kuo T, Chen L, Ferrara KW (2013). Cardiac myocyte exosomes: stability, HSP60, and proteomics. Am J Physiol Heart Circ Physiol.

[25] Gupta S, Knowlton AA (2007). HSP60 trafficking in adult cardiac myocytes: role of the exosomal pathway. Am J Physiol Heart Circ Physiol.

[26] Genneback N, Hellman U, Malm L, Larsson G, Ronquist G, Waldenstrom A et al. Growth factor stimulation of cardiomyocytes induces changes in the transcriptional contents of secreted exosomes. J Extracell Vesicles. 2013; 2. 10.3402/jev.v2i0.20167 PMC3760655 24009898

[27] Garcia NA, Moncayo-Arlandi J, Sepulveda P, Diez-Juan A (2016). Cardiomyocyte exosomes regulate glycolytic flux in endothelium by direct transfer of GLUT transporters and glycolytic enzymes. Cardiovasc Res.

[28] Garcia NA, Ontoria-Oviedo I, Gonzalez-King H, Diez-Juan A, Sepulveda P (2015). Glucose starvation in cardiomyocytes enhances exosome secretion and promotes angiogenesis in endothelial cells. PLoS One.

[29] Wang X, Huang W, Liu G, Cai W, Millard RW, Wang Y (2014). Cardiomyocytes mediate anti-angiogenesis in type 2 diabetic rats through the exosomal transfer of miR-320 into endothelial cells. J Mol Cell Cardiol.

[30] Wu K, Yang Y, Zhong Y, Ammar HM, Zhang P, Guo R (2016). The effects of microvesicles on endothelial progenitor cells are compromised in type 2 diabetic patients via downregulation of the miR-126/VEGFR2 pathway. Am J Physiol Endocrinol Metab.

[31] Kapustin AN, Chatrou ML, Drozdov I, Zheng Y, Davidson SM, Soong D (2015). Vascular smooth muscle cell calcification is mediated by regulated exosome secretion. Circ Res.

[32] Riquelme JA, Westermeier F, Hall AR, Vicencio JM, Pedrozo Z, Ibacache M et al. Dexmedetomidine protects the heart against ischemia-reperfusion injury by an endothelial eNOS/NO dependent mechanism. Pharmacol Res. 2015. 10.1016/j.phrs.2015.11.004 26607864

[33] Mittelbrunn M, Vicente-Manzanares M, Sanchez-Madrid F (2015). Organizing polarized delivery of exosomes at synapses. Traffic.

[34] Zhang L, Wrana JL (2014). The emerging role of exosomes in Wnt secretion and transport. Curr Opin Genet Dev.

[35] Yellon DM, Davidson SM (2014). Exosomes: nanoparticles involved in cardioprotection?. Circ Res.

[36] Mayo JN, Bearden SE (2015). Driving the hypoxia-inducible pathway in human pericytes promotes vascular density in an exosome-dependent manner. Microcirculation.

[37] Hergenreider E, Heydt S, Treguer K, Boettger T, Horrevoets AJ, Zeiher AM (2012). Atheroprotective communication between endothelial cells and smooth muscle cells through miRNAs. Nat Cell Biol.

[38] van Balkom BW, de Jong OG, Smits M, Brummelman J, den Ouden K, de Bree PM (2013). Endothelial cells require miR-214 to secrete exosomes that suppress senescence and induce angiogenesis in human and mouse endothelial cells. Blood.

[39] Liu R, Shen H, Ma J, Sun L, Wei M (2016). Extracellular vesicles derived from adipose mesenchymal stem cells regulate the phenotype of smooth muscle cells to limit intimal hyperplasia. Cardiovasc Drugs Ther.

[40] Lee C, Mitsialis SA, Aslam M, Vitali SH, Vergadi E, Konstantinou G (2012). Exosomes mediate the cytoprotective action of mesenchymal stromal cells on hypoxia-induced pulmonary hypertension. Circulation.

[41] Aliotta JM, Pereira M, Wen S, Dooner MS, Del Tatto M, Papa E (2016). Exosomes induce and reverse monocrotaline-induced pulmonary hypertension in mice. Cardiovasc Res.

[42] Madonna R, Van Laake LW, Davidson SM, Engel FB, Hausenloy DJ, Lecour S et al. Position paper of the European Society of Cardiology Working Group Cellular Biology of the heart: cell-based therapies for myocardial repair and regeneration in ischemic heart disease and heart failure. Eur Heart J. 2016. 10.1093/eurheartj/ehw113 PMC4912026 27055812

[43] Kishore R, Khan M (2016). More than tiny sacks: stem cell exosomes as cell-free modality for cardiac repair. Circ Res.

[44] Sahoo S, Klychko E, Thorne T, Misener S, Schultz KM, Millay M (2011). Exosomes from human CD34(+) stem cells mediate their proangiogenic paracrine activity. Circ Res.

[45] Mackie AR, Klyachko E, Thorne T, Schultz KM, Millay M, Ito A (2012). Sonic hedgehog-modified human CD34+ cells preserve cardiac function after acute myocardial infarction. Circ Res.

[46] Khan M, Nickoloff E, Abramova T, Johnson J, Verma SK, Krishnamurthy P (2015). Embryonic stem cell-derived exosomes promote endogenous repair mechanisms and enhance cardiac function following myocardial infarction. Circ Res.

[47] Barile L, Lionetti V, Cervio E, Matteucci M, Gherghiceanu M, Popescu LM (2014). Extracellular vesicles from human cardiac progenitor cells inhibit cardiomyocyte apoptosis and improve cardiac function after myocardial infarction. Cardiovasc Res.

[48] Ong SG, Lee WH, Huang M, Dey D, Kodo K, Sanchez-Freire V (2014). Cross talk of combined Gene and Cell therapy in ischemic heart disease: role of exosomal MicroRNA transfer. Circulation.

[49] Vrijsen KR, Sluijter JP, Schuchardt MW, van Balkom BW, Noort WA, Chamuleau SA (2010). Cardiomyocyte progenitor cell-derived exosomes stimulate migration of endothelial cells. J Cell Mol Med.

[50] Ibrahim AG, Cheng K, Marban E (2014). Exosomes as critical agents of cardiac regeneration triggered by cell therapy. Stem Cell Reports.

[51] Vandergriff AC, de Andrade JB, Tang J, Hensley MT, Piedrahita JA, Caranasos TG (2015). Intravenous cardiac stem cell-derived exosomes ameliorate cardiac dysfunction in doxorubicin induced dilated cardiomyopathy. Stem Cells Int.

[52] Tseliou E, Fouad J, Reich H, Slipczuk L, de Couto G, Aminzadeh M (2015). Fibroblasts rendered Antifibrotic, Antiapoptotic, and angiogenic by priming with cardiosphere-derived extracellular membrane vesicles. J Am Coll Cardiol.

[53] Bromage DI, Davidson SM, Yellon DM (2014). Stromal derived factor 1alpha: a chemokine that delivers a two-pronged defence of the myocardium. Pharmacol Ther.

[54] Lai RC, Arslan F, Lee MM, Sze NS, Choo A, Chen TS (2010). Exosome secreted by MSC reduces myocardial ischemia/reperfusion injury. Stem Cell Res.

[55] Arslan F, Lai RC, Smeets MB, Akeroyd L, Choo A, Aguor EN (2013). Mesenchymal stem cell-derived exosomes increase ATP levels, decrease oxidative stress and activate PI3K/Akt pathway to enhance myocardial viability and prevent adverse remodeling after myocardial ischemia/reperfusion injury. Stem Cell Res.

[56] Lai RC, Arslan F, Tan SS, Tan B, Choo A, Lee MM (2010). Derivation and characterization of human fetal MSCs: an alternative cell source for large-scale production of cardioprotective microparticles. J Mol Cell Cardiol.

[57] Sluijter JP, Condorelli G, Davidson SM, Engel FB, Ferdinandy P, Hausenloy DJ (2014). Novel therapeutic strategies for cardioprotection. Pharmacol Ther.

[58] Giricz Z, Varga ZV, Baranyai T, Sipos P, Paloczi K, Kittel A (2014). Cardioprotection by remote ischemic preconditioning of the rat heart is mediated by extracellular vesicles. J Mol Cell Cardiol.

[59] Lener T, Gimona M, Aigner L, Borger V, Buzas E, Camussi G (2015). Applying extracellular vesicles based therapeutics in clinical trials - an ISEV position paper. J Extracell Vesicles.

[60] Lodish H, Berk A, Zipursky SL, Matsudaira P, Baltimore D, Darnell J. Molecular cell biology. 4th edition. New York: 2000.

